# An efficient reversible privacy-preserving data mining technology over data streams

**DOI:** 10.1186/s40064-016-3095-3

**Published:** 2016-08-24

**Authors:** Chen-Yi Lin, Yuan-Hung Kao, Wei-Bin Lee, Rong-Chang Chen

**Affiliations:** 1 Department of Information Management, National Taichung University of Science and Technology, Taichung, Taiwan; 2 Department of Information Engineering and Computer Science, Feng Chia University, Taichung, Taiwan; 3 Department of Distribution Management, National Taichung University of Science and Technology, Taichung, Taiwan

**Keywords:** Cloud computing, Data streams, Sliding window, Data protection

## Abstract

With the popularity of smart handheld devices and the emergence of cloud computing, users and companies can save various data, which may contain private data, to the cloud. Topics relating to data security have therefore received much attention. This study focuses on data stream environments and uses the concept of a sliding window to design a reversible privacy-preserving technology to process continuous data in real time, known as a continuous reversible privacy-preserving (CRP) algorithm. Data with CRP algorithm protection can be accurately recovered through a data recovery process. In addition, by using an embedded watermark, the integrity of the data can be verified. The results from the experiments show that, compared to existing algorithms, CRP is better at preserving knowledge and is more effective in terms of reducing information loss and privacy disclosure risk. In addition, it takes far less time for CRP to process continuous data than existing algorithms. As a result, CRP is confirmed as suitable for data stream environments and fulfills the requirements of being lightweight and energy-efficient for smart handheld devices.

## Background

With the rapid advances in network technologies and the popularity of smart handheld devices, user habits are gradually changing. Cloud computing is the emerging concept in response to these changes. The objective of cloud computing is to provide better services through the Internet and network computing so that computers can collaborate with each other and share hardware and software resources and data (Kshetri 2013).

In recent years, users and companies have been collecting and saving various data to the cloud, which they analyse to find hidden business opportunities. Owing to the enormous volume of data in a cloud environment, it is impossible to use a manual process to sort the data into the information that can be understood by people. Data mining based on big data has become an important service for cloud computing (Wu et al. 2014; Jiang and Liu 2015; Chen et al. 2014). However, big data may contain users’ and companies’ private data, presenting a need data security (Chen et al. 2014; Zhang et al. 2014; Singh et al. 2014; Bianchi et al. 2009). For example, a user uses a smart handheld device to regularly access physiological data including her/his heartbeat, blood pressure, blood glucose, and oxygen content, as shown in Table 1. After accessing these data, they are automatically saved to the cloud. Data companies can analyse the physiological data in the cloud and monitor the health status of the user. However, the data in the cloud could be tampered with by hackers, putting the integrity of the data into question. In addition, the data collection companies could pass the user’s personal data to a third party without the user’s consent, which causes concerns regarding the disclosure of the user’s private data. To prevent this from happening, users can perform privacy preserving to the original data and save the protected data to the cloud. Data companies can use data mining techniques to analyse the protected data in the cloud. The analysed results of the protected data should be similar to that of the original data. This type of research is known as Privacy Preserving Data Mining (PPDM; Hao et al. 2011; Hajian et al. 2014; Chun et al. 2013; Yang and Qiao 2010; Zhu et al. 2009; Herranz et al. 2010).

**Table 1:** Pathology information

Time	Heartbeat	Blood pressure	Blood glucose	Oxygen content
1	77	145	125	170
2	75	148	121	167
3	76	147	123	169
4	78	146	122	168
5	77	147	124	170
6	76	146	123	171
7	77	145	125	169
8	75	147	126	170
9	76	148	124	168
10	75	149	125	169
11	76	148	124	171
12	78	147	128	170
…	…	…	…	…
…	…	…	…	…
…	…	…	…	…

The objective of PPDM is to effectively protect private information while retaining the knowledge contained in the original data. It often uses methods such as swap (Chun et al. 2013; Zhu et al. 2009), modification (Yang and Qiao 2010; Zhu et al. 2009) and deletion (Herranz et al. 2010) operations to protect the original data so that no correlation exists between the original data and the resultant protected data. As a result, it cannot recover the original data from the protected data. If the original data is lost, the users cannot verify the authenticity of the protected data, which then creates issues with knowledge uncertainty (Herranz et al. 2010; Chen et al. 2013; Hong et al. 2010).

To solve the inability of PPDM to recover the original data from the protected data, which causes issues with knowledge uncertainty, Chen et al. (2013) used the concept of reversible data hiding (Zhang et al. 2014; Chang et al. 2014; Hong and Chen 2012; Tian 2003) in image processing and proposed the privacy difference expansion (PDE) algorithm, which is capable of protecting and recovering the original data. In addition to preserving the advantages of PPDM, the PDE algorithm allows users to embed a customised watermark while protecting the original data. When the users are concerned about the protected data, the protected data can be recovered and the watermark can be extracted. The user compares the watermark with their customised watermark to verify the integrity of the protected data. For the data protection phase, the PDE algorithm uses the difference between the successive values in the original data to determine the corresponding values in the protected data. When the difference between the successive values in the original data is larger, the difference between the corresponding values in the protected data is also larger. As a result, the content of the original data and the content of the protected data are more dissimilar, which makes that the knowledge retained in the protected data is reduced. To minimise this reduction, before the data protection is performed on the original data, the PDE algorithm first performs a principal component analysis (PCA; Abdi and Williams 2010) of the original data, so that data with smaller differences can be included in the process. Using Table 1 as an example, users’ physiological data changes with the time, and the data storage for the smart handheld devices is limited; therefore, as soon as the physiological data is accessed, the data needs to be protected and saved to the cloud within a short period. However, to retain the knowledge saved in the protected data as much as possible, the PDE algorithm must use PCA to determine the sequence of data protection after all the data has been collected. Therefore, the requirement for real-time data processing cannot be fulfilled.

Based on the above, this study uses the concept of a sliding window and designs a privacy-preserving technology to process continuous data in real time, targeting a data stream environment. The algorithm, called the continuous reversible privacy-preserving (CRP) algorithm, can recover the protected data and extract the embedded watermark to verify whether the protected data has been tampered with. The requirements for protecting private information and performing data mining of the continuous data can be fulfilled at the same time. The details are explained in the following sections.

## Continuous reversible privacy-preserving (CRP) algorithm

To protect the content in the data in a data stream environment while meeting the requirements for data mining, we use the concept of a sliding window and propose the CRP algorithm, which can effectively process this type of data. The algorithm has a data protection phase and a data recovery phase. In the data protection phase, a sliding window model is used to protect the content in the continuous data, and the watermarks customised by users are embedded. On the other hand, in the data recovery phase, in addition to recovering the original data from the protected data, one can also extract the embedded watermark to verify whether the protected data has been tampered with. The data protection and recovery phases in CRP are detailed below.

### Data protection phase

**Table:** 

Input:	A window size *s*, a user-defined watermark *w*, and streaming data *D* = {*d*_1_, *d*_2_, *d*_3_, …} (*d*_*i*_ = (*d*_*i*,1_, *d*_*i*,2_, *d*_*i*,3_, …, *d*_*i*,*m*_), *i* ≥ 1, *m* is the number of the attributes in *D*).
Output:	The protected data $D^{{\prime }} = \{ d_{1}^{{\prime }} ,d_{2}^{{\prime }} ,d_{3}^{{\prime }} , \ldots \}$.
*Step 1*.	Let *p* = 1, *l* = 1.
*Step 2*.	Let $d_{1}^{{\prime }} = d_{1}$, $d_{2}^{{\prime }} = d_{2}$, …, and $d_{(p + s - 1)}^{{\prime }} = d_{(p + s - 1)}$, save $d_{1}^{{\prime }} ,d_{2}^{{\prime }} , \ldots ,$ and $d^{\prime}_{(p + s - 1)}$ to the cloud.
*Step 3*.	For (*j* = 1; *j* <= *m*; *j*++)
	Let *avg* = $\left\lfloor {(d_{p,j}^{{\prime }} + d_{p + 1,j}^{{\prime }} + \cdots + d_{(p + s - 1),j}^{{\prime }} )/s} \right\rfloor$, and *diff* = *d*_*p*+*s,j*_ − *avg*.
	**// Protecting the value of** ***d***_***p*+*s,j***_
	If *diff* > 1 Then $d_{p + s,j}^{{\prime }} = d_{p + s,j} + 1$.
	Else If *diff* < 0 Then $d_{p + s,j}^{{\prime }} = d_{p + s,j} - 1$.
	**// Embedding watermark** ***w***
	If *l* < |*w*|
	If *diff* == 0 Then {$d_{p + s,j}^{{\prime }} = d_{p + s,j}$ – the *l* ^th^ bit of the watermark *w*, *l* = *l* + 1}.
	Else If *diff* == 1 Then {$d_{p + s,j}^{{\prime }} = d_{p + s,j}$ + the *l* ^th^ bit of the watermark *w*, *l* = *l* +1}.
*Step 4*.	Save $d_{(p + s)}^{{\prime }}$ to the cloud, *p* = *p* + 1, and go to *Step 3*.

**Fig. 1:**
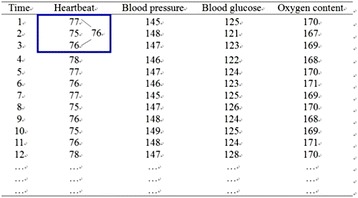
Schematic diagram of CRP data protection

The example in Table 1 explains the data protection phase of CRP. Assume the window size *s* is 3, and the watermark *w* is (0000111101001…)_2_. First, since the window size *s* is 3, the current sliding window has the first three rows of data. According to *Step 2*, these data in the window are not required to go through the protection process and can be directly saved to the cloud. In *Step 3*, the average value for Heartbeat in the sliding window is calculated to be 76 $\left( { = \left\lfloor {(77 + 75 + 76)/3} \right\rfloor } \right)$, as shown in the frame in Fig. 1. Subtracting 76 from the fourth value 78 of Heartbeat, the value for *diff* is 2 (= 78 − 76). Since *diff* = 2, the fourth value in Heartbeat after data protection is 79 (= 78 + 1). Next, using a similar method, the fourth values in blood pressure, blood glucose, and oxygen content after data protection are calculated and saved to the cloud. The sliding window then slides to the second, third and fourth rows. The above method is used to obtain the values after data protection of the fifth row. Table 2 shows the data after protection.

**Table 2:** CRP data protected by generalising Table 1

Time	Heartbeat	Blood pressure	Blood glucose	Oxygen content
1	77	145	125	170
2	75	148	121	167
3	76	147	123	169
4	79	146	121	168
5	77	147	125	171
6	75	145	122	172
7	77	144	126	168
8	74	148	127	169
9	76	149	123	167
10	74	150	125	169
11	77	147	123	172
12	79	146	129	171
…	…	…	…	…
…	…	…	…	…
…	…	…	…	…

### Data recovery phase

After the data protection, if users have concerns about the protected data, the protected data can be recovered and the watermark can be extracted to compare whether the watermark is the same as the watermark configured by the users. This is to verify the integrity of the protected data. The process for the data recovery phase is shown below.

**Table:** 

Input:	The window size *s*, and the protected data $D^{{\prime }} = \{ d_{1}^{{\prime }} ,d_{2}^{{\prime }} ,d_{3}^{{\prime }} , \ldots \}$ ($d_{i}^{{\prime }} = (d_{i,1}^{{\prime }} ,d_{i,2}^{{\prime }} ,d_{i,3}^{{\prime }} , \ldots ,d_{i,m}^{{\prime }}$), *i* ≥ 1, *m* is the number of the attributes in $D^{{\prime }}$).
Output:	The original streaming data *D* = {*d*_1_, *d*_2_, *d*_3_, …}, and the user-defined watermark *w*.
*Step 1*.	Let *p* = 1, *l* = 1.
*Step 2*.	Let $d_{1} = d_{1}^{{\prime }}$, $d_{2} = d_{2}^{{\prime }}$, …, and $d_{(p + s - 1)} = d_{(p + s - 1)}^{{\prime }}$.
*Step 3*.	For (*j* = 1; *j* <= *m*; *j*++)
	Let *avg* = $\left\lfloor {(d_{p,j}^{{\prime }} + d_{p + 1,j}^{{\prime }} + \cdots + d_{(p + s - 1),j}^{{\prime }} )/s} \right\rfloor$, and *diff* = $d_{p + s,j}^{{\prime }}$ − *avg*.
	**// Recovering the value of** ***d***_***p*+*s,j***_ **and decoding watermark** ***w***
	If *diff* == 0 or *diff* == 1 Then {the *l* ^th^ bit of the watermark *w* is (0)_2_, $d_{p + s,j} = d^{\prime}_{p + s,j}$, *l* = *l* + 1}.
	Else If *diff* == –1 Then {the *l* ^th^ bit of the watermark *w* is (1)_2_, $d_{p + s,j} = d_{p + s,j}^{{\prime }} + 1$, *l* = *l* + 1}.
	Else If *diff* == 2 Then {the *l* ^th^ bit of the watermark *w* is (1)_2_, $d_{p + s,j} = d_{p + s,j}^{{\prime }} - 1$, *l* = *l* + 1}.
	Else If *diff* > 2 Then {$d_{p + s,j} = d_{p + s,j}^{{\prime }} - 1$}
	Else If *diff* < –1 Then {$d_{p + s,j} = d_{p + s,j}^{{\prime }} + 1$}.
*Step 4*.	*p* = *p* + 1, and go to *Step 3*.

Table 2 is used as an example to explain the data recovery phase of CRP. Assuming the window size *s* is 3, the current sliding window has the first three rows of data. According to *Step 2*, these data are not required to go through the recovery process and are thus directly configured as the recovered data. In *Step 3*, the average value for Heartbeat in the sliding window is calculated to be 76 $\left( { = \left\lfloor {(77 + 75 + 76)/3} \right\rfloor } \right)$. Subtracting the value 76 from the fourth value 79 of Heartbeat, the value for *diff* is 3 (= 79 − 76). For the fourth value of Heartbeat, the value after data recovery is 78 (= 79 − 1). Next, using a similar method, the values for Blood pressure, Blood glucose, and Oxygen content after data recovery for the fourth values of data are calculated. Then, the sliding window will slide to the second, third and fourth rows of data. The above method is then used to obtain the value after data recovery of the fifth row of data. Table 2 shows that, when all the data have gone through the data recovery process, the results are the same as the original data in Table 1, and the watermark *w* is (0000111101001…)_2_.

## Measures

In the experiment, we use the assessment method for measuring (Herranz et al. 2010; Chen et al. 2013; Mateo-Sanz et al. 2005) the effectiveness of PPDM to assess the performance of the CRP algorithm. The concept of the assessment method is explained in this section.

The objective of PPDM is to reduce the disclosure risk of the privacy in the original data while considering the value of data mining for the protected data (Hao et al. 2011; Hajian et al. 2014; Chun et al. 2013; Yang and Qiao 2010; Zhu et al. 2009; Herranz et al. 2010; Chen et al. 2013). In order to protect the privacy in the original data, data protection methods often cause information loss, and protected data can therefore lose the value of data mining (Mateo-Sanz et al. 2005; Herranz et al. 2012). An important basis for analysing the effectiveness of PPDM protection is achieving a balance between knowledge reservation, information loss, and privacy disclosure risk (Herranz et al. 2010; Chen et al. 2013).

In terms of knowledge reservation, our assessment is usually based on the classification used in financial and medical estimations (Zhu et al. 2009; Chang et al. 2010; Chiu et al. 2014). Three well-known classifiers, namely Decision Tree, Native Bayes, and Support Vector Machine (SVM), in conjunction with 10-fold cross validation (Chen et al. 2013; Chiu et al. 2014), are used to analyse the impact of CRP on knowledge reservation. In addition, we used Probabilistic Information Loss (PIL; Herranz et al. 2010; Chen et al. 2013) proposed by Mateo-Sanz et al. (2005) to assess the degree of information loss of protected data. PIL calculates the mean, variance, covariance, Pearson’s correlation, and quantiles before and after the data protection. Using data standardisation, the range of values for statistical analysis is limited to [0,1], and the difference before and after the data protection is expressed as a percentage. A smaller PIL value means a smaller degree of information loss. Disclosure Risk (DR; Herranz et al. 2010; Chen et al. 2013; Herranz et al. 2012) combines the two similarity calculation methods, interval disclosure (ID) and distance linkage disclosure (DLD), to assess the disclosure risk of private data in the protected data. ID is for calculating whether the attribute values of the protected data have the same ratio as the attribute values of the corresponding original data. DLD uses Euclidean distance to assess the similarities in data and to calculate if the same data exists in the original and protected data. DLD also records the ratio of identical data to show the degree of similarity between the protected data and the original data. The configuration for extension in our experiments, i.e. *DR* = 0.5 × *ID* + 0.5 × *DLD* (Herranz et al. 2010; Chen et al. 2013; Herranz et al. 2012), was used to assess the privacy disclosure risk. A smaller DR value indicates a smaller privacy disclosure risk.

## Experimental results

In our experiments, five test datasets included in UCI Machine Learning Repository (Frank and Asuncion 2012) and the U.S. Census Bureau (2012) was used to show the performance of the CRP algorithm; the detailed information of the test datasets is summarised in Table 3. In addition, we compared the CRP algorithm with the PDE algorithm. In order to retain the knowledge of the original data as much as possible, the PDE algorithm used PCA to determine the data processing sequence. Lastly, three types of measures including classification analysis, PIL, and DR are used to compare the performance of CRP with that of PDE. In this paper, the test platform is equipped with Intel Core i5 2.67 GHz, 4 GB of memory and Windows 7 Professional 64 bit Operating System. Java was used to implement the CRP and PDE algorithms.

**Table 3:** Test datasets

Datasets name	Number of attributes	Number of instances	Number of classes
Abalone	8	4177	3
Breast Cancer Wisconsin (original)	10	699	2
Census	12	13,518	5
Landsat Satellite	36	4435	7
Vehicle Silhouettes	18	846	4

**Fig. 2:**
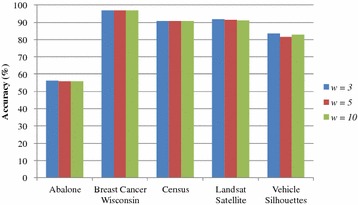
Analysis of window sizes on SVM

Figure 2 shows the relationship between the knowledge accuracy evaluated by SVM and window sizes. From the figure, it is obtained that the sliding window sizes have no obvious effect on the knowledge accuracy of the test datasets with CRP protection. The reason is that no matter what the window sizes are, the difference between the original value and the corresponding protected value has to be 1, 0, or −1 according to the value *diff*. As a result, the content of the original data and that of the protected data are similar.

In the following experiments, the default value for the sliding window size is set to be 3 based on experimental observations.

**Fig. 3:**
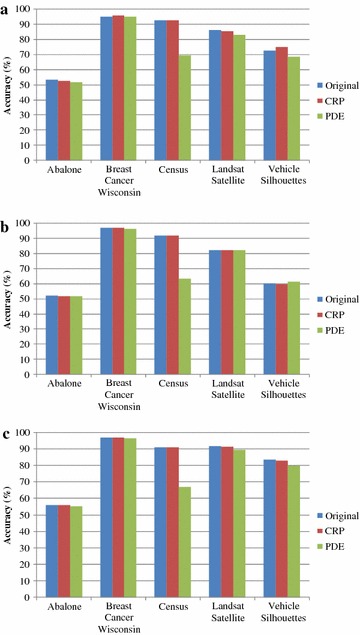
Analysis of knowledge accuracy. **a** Decision Tree, **b** Native Bayes, **c** SVM

In Fig. 3, three classifiers, namely Decision Tree, Native Bayes, and SVM, are used for classifying knowledge accuracy analysis on test datasets with the CRP and PDE algorithm protection. In Fig. 3a, the test results from Decision Tree show that the knowledge accuracy of the test datasets with CRP protection are close to the knowledge accuracy of the original data, indicating that the test datasets with CRP protection retained their original knowledge. However, the knowledge accuracy of the test datasets Census, Landsat Satellite, and Vehicle Silhouettes with PDE protection is reduced by 23.23, 3.07, and 4.02 %, respectively, indicating that data with PDE protection can affect the knowledge exploration. Native Bayes in Fig. 3b and SVM in Fig. 3c show that the accuracy of test datasets with CRP protection are close to the accuracy of the original data, with a difference of less than 1 %. However, in Fig. 3b, c, the accuracy of the Census dataset with PDE protection is reduced by 28.48 and 23.91 %, respectively. In addition, in Fig. 3c, the Vehicle Silhouettes dataset with PDE protection is also reduced by 3.93 %. Based on the above classification knowledge analysis, in regards to Decision Tree, Native Bayes, and SVM, the knowledge accuracy for the test datasets with CRP algorithm protection are close to the knowledge accuracy of the original test datasets. However, for the PDE algorithm, the accuracy is reduced because the original datasets have been extensively modified. This shows that the CRP algorithm is very effective in terms of knowledge reservation and is better than the PDE algorithm.

**Fig. 4:**
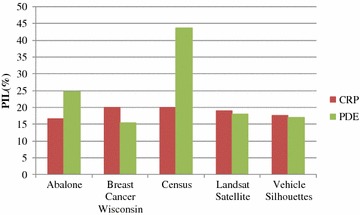
Analysis of PIL values

**Fig. 5:**
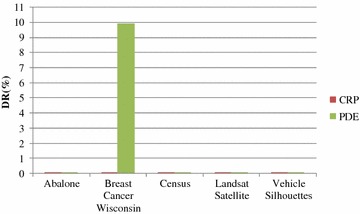
Analysis of DR values

Figures 4 and 5 show the PIL and DR test results for CRP and PDE, respectively. Figure 4 shows that, for datasets with CRP protection, the PIL values are below 20 %. For PDE, even though the PIL values in three datasets are lower than those for CRP, the PIL values for datasets with PDE protection are generally unstable. The PIL values for Abalone and Census datasets are greater than 20 %. It can be observed that CRP can indeed reduce information loss effectively, and compared to that of PDE, CRP has more stable results for PIL. DR analysis in Fig. 5 shows that the DR values of CRP are all lower than 0.02 %, confirming that CRP can effectively lower privacy disclosure risk. In addition, with regards to DR, CRP shows a better performance and is more stable than PDE.

**Fig. 6:**
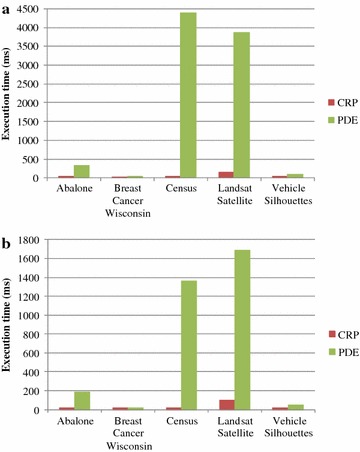
Analysis of execution time. **a** Data protection, **b** data recovery

To show the effectiveness of CRP in regards to the continuous data process, we calculated the execution time required for CRP to protect and recover data; the results are shown in Fig. 6. It can be observed that, from these five test datasets, the execution times for CRP to perform data protection and data recovery were less than 140 ms, which is far less than the execution times required by PDE, especially for the Census and Landsat Satellite datasets. Thus, CRP can effectively protect private data and is valuable for data mining. The time cost for continuous data processing is also far less than that of PDE. As a result, CRP is suitable for using in a data stream environment and meets the requirements of being lightweight and energy-efficient for smart handled devices.

## Conclusions

With the popularity of smart handheld devices and the emergence of cloud computing, users and companies are saving various data to the cloud. Data companies collect data and perform data analysis to find hidden business opportunities. These data may contain private data, making a serious issue data security. To protect private data, our study targeted a data stream environment and designed the CRP algorithm based on the sliding window model to protect continuous data. After CRP protection, the original data can be accurately restored using the data recovery program. In addition, data integrity can be verified using an embedded watermark. For our experiments, we used data for knowledge reservation, information loss, and privacy disclosure risk to assess the effectiveness of CRP. The processing times required for data protection and data recovery were calculated. The experiment results confirmed that, from knowledge reservation and privacy disclosure risk analysis, CRP had better performance than the existing PDE algorithm. In terms of information loss, CRP also had better stability than PDE. In addition, CRP required less execution time than the PDE algorithm in regards to data protection and data recovery, indicating that CRP can effectively protect the privacy in continuous data and can also meet the requirements for knowledge analysis; it is therefore suitable for a cloud computing environment and smart handheld devices.

## References

[CR1] Abdi H, Williams LJ (2010). Principal component analysis. Comput Stat.

[CR2] Bianchi T, Piva A, Barni M (2009). On the implementation of the discrete Fourier transform in the encrypted domain. IEEE Trans Inf Forensics Secur.

[CR3] Chang HY, Chiou CJ, Chen NS (2010). Impact of mental health and caregiver burden on family caregivers’ physical health. Arch Gerontol Geriatr.

[CR4] Chang CC, Nguyen TS, Lin CC (2014). Reversible data embedding for indices based on histogram analysis. J Vis Commun Image Represent.

[CR5] Chen TS, Lee WB, Chen J, Kao YH, Hou PW (2013). Reversible privacy preserving data mining: a combination of difference expansion and privacy preserving. J Supercomput.

[CR6] Chen M, Mao S, Liu Y (2014). Big data: a survey. Mob Netw Appl.

[CR7] Chiu CC, Yeh SJ, Hu YH, Liao KYK (2014). SVM classification for diabetics with various degrees of autonomic neuropathy based on cross-correlation features. J Med Biol Eng.

[CR8] Chun JY, Hong D, Jeong IR, Lee DH (2013). Privacy-preserving disjunctive normal form operations on distributed sets. Inf Sci.

[CR9] Frank A, Asuncion A (2012) UCI machine learning repository. http://archive.ics.uci.edu/ml/. Accessed 6 Sept 2012

[CR10] Hajian S, Domingo-Ferrer J, Farràs O (2014). Generalization-based privacy preservation and discrimination prevention in data publishing and mining. Data Min Knowl Disc.

[CR11] Hao Z, Zhong S, Yu N (2011). A privacy-preserving remote data integrity checking protocol with data dynamics and public verifiability. IEEE Trans Knowl Data Eng.

[CR12] Herranz J, Matwin S, Nin J, Torra V (2010). Classifying data from protected statistical datasets. Comput Secur.

[CR13] Herranz J, Nin J, Solé M (2012). Kd-trees and the real disclosure risks of large statistical databases. Inf Fusion.

[CR14] Hong W, Chen TS (2012). A novel data embedding method using adaptive pixel pair matching. IEEE Trans Inf Forensics Secur.

[CR15] Hong TP, Tseng LH, Chien BC (2010). Mining from incomplete quantitative data by fuzzy rough sets. Expert Syst Appl.

[CR16] Jiang P, Liu XS (2015). Big data mining yields novel insights on cancer. Nat Genet.

[CR17] Kshetri N (2013). Privacy and security issues in cloud computing: the role of institutions and institutional evolution. Telecommun Policy.

[CR18] Mateo-Sanz JM, Domingo-Ferrer J, Sebé F (2005). Probabilistic information loss measures in confidentiality protection of continuous microdata. Data Min Knowl Disc.

[CR19] Singh K, Guntuku SC, Thakur A, Hota C (2014). Big data analytics framework for peer-to-peer botnet detection using random forests. Inf Sci.

[CR20] Tian J (2003). Reversible data embedding using a difference expansion. IEEE Trans Circuits Syst Video Technol.

[CR21] U.S. Census Bureau (2012) Census Bureau home page. http://www.census.gov/. Accessed 3 Sept 2012

[CR22] Wu X, Zhu X, Wu GQ, Ding W (2014). Data mining with big data. IEEE Trans Knowl Data Eng.

[CR23] Yang W, Qiao S (2010). A novel anonymization algorithm: privacy protection and knowledge preservation. Expert Syst Appl.

[CR24] Zhang X, Qian Z, Feng G, Ren Y (2014). Efficient reversible data hiding in encrypted images. J Vis Commun Image Represent.

[CR25] Zhu D, Li XB, Wu S (2009). Identity disclosure protection: a data reconstruction approach for privacy-preserving data mining. Decis Support Syst.

